# Peniciadametizine A, a Dithiodiketopiperazine with a Unique Spiro[furan-2,7*'*-pyrazino[1,2-*b*][1,2]oxazine] Skeleton, and a Related Analogue, Peniciadametizine B, from the Marine Sponge-Derived Fungus *Penicillium adametzioides*

**DOI:** 10.3390/md13063640

**Published:** 2015-06-05

**Authors:** Yang Liu, Attila Mándi, Xiao-Ming Li, Ling-Hong Meng, Tibor Kurtán, Bin-Gui Wang

**Affiliations:** 1 Key Laboratory of Experimental Marine Biology, Institute of Oceanology, Chinese Academy of Sciences, Nanhai Road 7, Qingdao 266071, China; E-Mails: buckuper@163.com (Y.L.); lixmqd@aliyun.com (X.-M.L.); m8545303@163.com (L.-H.M.); 2 University of Chinese Academy of Sciences, Yuquan Road 19A, Beijing 100049, China; 3 Department of Organic Chemistry, University of Debrecen, P.O. Box 20, Debrecen 4010, Hungary; E-Mail: mandia@delfin.klte.hu

**Keywords:** marine sponge, endophytic fungus, *Penicillium adametzioides*, secondary metabolites, antifungal activity

## Abstract

Peniciadametizine A (1); a new dithiodiketopiperazine derivative possessing a unique spiro[furan-2,7*'*-pyrazino[1,2-*b*][1,2]oxazine] skeleton, together with a highly oxygenated new analogue, peniciadametizine B (2); as well as two known compounds, brasiliamide A (3); and viridicatumtoxin (4), were isolated and identified from *Penicillium adametzioides* AS-53, a fungus obtained from an unidentified marine sponge. The unambiguous assignment of the relative and absolute configuration for the spiro center C-2 of compound 1 was solved by the combination of NMR and ECD measurements with Density-Functional Theory (DFT) conformational analysis and Time-Dependent Density-Functional Theory-Electronic Circular Dichroism (TDDFT-ECD) calculations. The spiro[furan-2,7*'*-pyrazino[1,2-*b*][1,2]oxazine] skeleton of **1** has not been reported yet among natural products and the biosynthetic pathway for **1** and **2** was discussed. Compounds **1** and **2** showed inhibitory activity against the pathogenic fungus *Alternaria brassicae*.

## 1. Introduction

**Figure 1:**
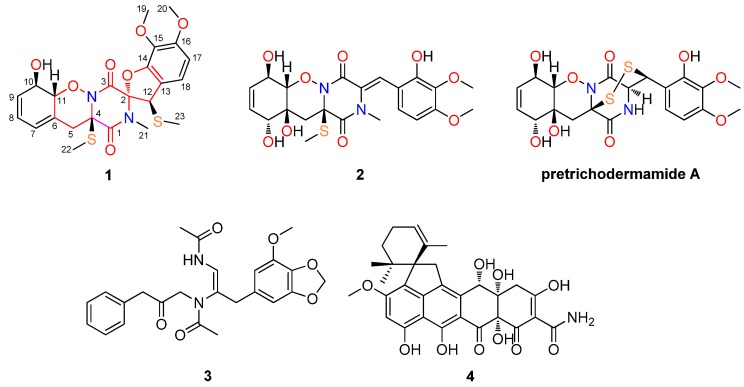
Structures of the isolated compounds **1**–**4** and reference compound pretrichodermamide A.

The dithiodiketopiperazine derivatives represent a unique class of fungal metabolites generally possessing a disulfide bridge or having two methylthio groups, with the sulfur-atom usually attached to α-positions of the cyclic dipeptide [1]. In contrast, dithiodiketopiperazines with the disulfide functionality attached to both α- and β-positions of the amino acid residues, such as gliovirin [1] and aspirochlorine [2], are reported very rarely. A peculiar structural feature of these two compounds is the presence of an *O*-alkyl-oxime or a spiro[furan-pyrazino] functionality, respectively [1,2,3]. In the last two decades, marine filamentous fungi have been recognized as an important source of structurally unusual and biologically active natural products [4]. During our ongoing search for bioactive metabolites from marine-derived fungi [5,6,7,8,9], we have recently reported a new spiroquinazoline derivative, *N*-formyllapatin A, from *Penicillium adametzioides* AS-53, a fungus isolated from an unidentified sponge collected at the Hainan Island of China [7]. Futher exploration of the fungal extract resulted in the isolation and characterization of two new diketopiperazine analogues, peniciadametizines A and B (**1** and **2**); as well as two known compounds, brasiliamide A (3) [10]; and viridicatumtoxin (4) [11] (Figure 1). Among them, compound **1** is a dithiodiketopiperazine derivative possessing a unique spiro[furan-2,7*'*-pyrazino[1,2-*b*][1,2]oxazine] skeleton and the two sulfur atoms are attached to the α- and β-positions of the amino acid residues. This paper describes the isolation, structural elucidation, and antimicrobial activity of compounds **1**–**4**.

## 2. Results and Discussion

Peniciadametizine A (**1**), obtained as a colorless amorphous powder, had the molecular formula C_23_H_26_N_2_O_7_S_2_ on the basis of the positive HR-ESI-MS adduct ion [M + Na]^+^ at *m*/*z* 529.1060, implying the presence of 12 degrees of unsaturation. The IR spectrum exhibited broad absorption for OH groups (3334 cm^−1^) as well as for amide chromophores (1679 and 1602 cm^−1^). Inspection of the ^13^C NMR and DEPT spectroscopic data of **1** (Table 1) revealed the presence of five methyls (with two oxygenated, one nitrogenated, and two sulfurated), one methylene, eight methines (with five aromatic/olefinic and three sulfur/oxygenated sp^3^), and nine quaternary (with two amide carbonyl, five aromatic/olefinic, and two nitrogen/oxygenated/thiogenated sp^3^) carbons. The ^1^H NMR and HSQC data (Table 1) indicated the presence of 26 protons, attributable to five methyl singlets (H_3_-19 to H_3_-23), one aliphatic methylene (H_2_-5), and eight methines, with two aromatic (H-17 and H-18), three olefinic (H-7 to H-9), and three sulfur/oxygenated (H-10 to H-12), as well as an exchangeable OH group (10-OH).

**Table 1:** ^1^H-, ^13^C-NMR and HMBC data of compounds **1** and **2**.

No.	1 (Acquired in DMSO-*d*_6_)			2 (Acquired in Acetone-*d*_6_)		
	δ_C_^*a*^, Type	δ_H_ (*J* in Hz) ^*b*^	HMBC	δ_C_^*a*^, Type	δ_H_ (*J* in Hz) ^*b*^	HMBC
1	161.7, C			164.9, C		
2	100.8, C			129.0, C		
3	158.7, C			160.9, C		
4	70.4, C			68.1, C		
5	36.8, CH_2_	3.58, d (14.0)	1, 4, 6, 7, 11	33.0, CH_2_	2.09, d (15.0)	6, 7
		2.98, d (14.0)			2.54, d (15.0)	
6	129.7, C			74.0, C		
7	122.2, CH	5.82, d (2.5)	6, 8	75.2, CH	4.40, br s	6, 8
8	122.4, CH	5.76, dd (10.0, 2.5)	6, 10	130.3, CH	5.54, br d (10.4)	6, 10
9	132.2, CH	5.69, br d (10.0)	7, 11	128.9, CH	5.60, br d (10.4)	7, 11
10	69.5, CH	4.54, br dd (13.5, 4.5)		65.9, CH	4.65, br s	9
11	91.4, CH	4.92, d (13.5)	10	89.8, CH	4.00, d (8.2)	6, 10
12	53.5, CH	4.95, br s	2, 3, 13, 14, 23	118.1, CH	7.25, s	3, 14, 18
13	118.3, C			115.6, C		
14	149.7, C			150.1, C		
15	132.3, C			137.1, C		
16	153.1, C			154.8, C		
17	107.0, CH	6.76, d (8.4)	13, 15, 16	104.8, CH	6.66, d (8.7)	13, 15
18	119.2, CH	6.94, d (8.4)	12, 14, 16	126.0, CH	6.90, d (8.7)	14, 16
19	60.2, CH_3_	3.73, s	15	61.0, CH_3_	3.79, s	15
20	56.2, CH_3_	3.80, s	16	56.4, CH_3_	3.89, s	16
21	28.2, CH_3_	2.78, s	1, 2	35.6, CH_3_	2.91, s	1, 2
22	13.2, CH_3_	2.36, s	4	13.9, CH_3_	2.26, s	4
23	13.0, CH_3_	1.78, s	12			
10-OH		5.29, d (4.5)				

^*a*^ Measured at 125 MHz. ^*b*^ Measured at 500 MHz. Multiplicities were determined by DEPT and HSQC experiments.

**Figure 2:**
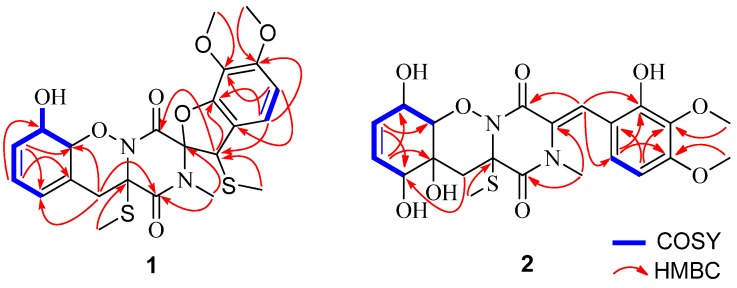
Key ^1^H–^1^H COSY and HMBC correlations of compounds **1** and **2**.

Detailed interpretation of the COSY and HSQC spectra of **1** resulted in the elucidation of two discrete proton-proton spin-coupling systems corresponding to a =CH–CH=CH–CH(OH)–CH– unit (I, C-7 through C-11) and an aromatic –CH=CH– residue (II, C-17 to C-18) (Figure 2). HMBC correlations from H-7, H-8 to C-6, from H_2_-5 to C-1, C-4, C-6, C-7, and C-11, as well as from H_3_-22 to C-4 indicated the involvement of an *O*-alkyl-oxime motif, thus affording half of the ketopiperazine ring in the molecule of **1**. The structure of the other half of the ketopiperazine ring was deduced by the HMBC correlations from H-12 to C-2, C-3, C-13, and C-14, from H-17 to C-13, C-15, and C-16, and from H-18 to C-12, C-14, and C-16. Furthermore, HMBC correlations from H_3_-19 to C-15, from H_3_-20 to C-16, from H_3_-21 to C-1 and C-2, and from H_3_-23 to C-12 verified the position of these methyl groups. Finally, the connection of C-2 and C-14 through an oxygen atom allowed the construction of the spiro[furan-pyrazino] functionality to match the molecular formula and the required degrees of unsaturation.

**Figure 3:**
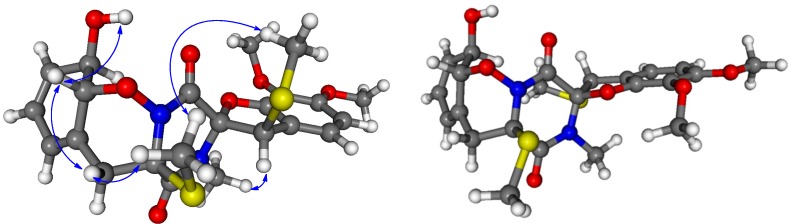
Measured NOE correlations of **1** shown on the lowest-energy B97D/TZVP computed conformer of (2*R*,4*R*,10*R*,11*R*,12*R*)-**1** and lowest-energy conformer of (2*S*,4*R*,10*R*,11*R*,12*R*)-**1**.

Analysis of vicinal proton-proton coupling constant and NOESY data enabled assignment of the relative configuration of **1**. The large ^3^*J* coupling constant of H-10 and H-11 (13.5 Hz) and the observed NOE correlation from the proton of 10-OH to H-11 established the *trans-*diaxial orientation of H-10 and H-11, whereas the observed NOE correlations from H_a_-5 to H-11 and H_3_-22 and from H_3_-22 to H_3_-23 placed these groups on the same side of the molecule (Figure 3). However, the NOESY spectrum lacked diagnostic correlations to unambiguously determine the relative configuration of the spiro carbon (C-2), although the NOE correlation from H-12 to H_3_-21 was observed in the NOESY spectrum. To solve this problem, conformational analyses were carried out on the diastereomeric (2*R*,4*R*,10*R*,11*R*,12*R*)-**1** and (2*S*,4*R*,10*R*,11*R*,12*R*)-**1**. In the B97D/TZVP (triple-zeta valence polarization) computed conformers of (2*R*,4*R*,10*R*,11*R*,12*R*)-**1**, differing mainly in the orientation of the methylthio and methoxy groups, the 1,2-oxazine ring adopted a boat conformation and the *N*-methyl protons and H-12 were on the same face at a distance of 2.68 Å (Figure 3). Similarly, the two methylthio groups were also on the same side in accordance with their observed NOE correlation. In contrast, in the computed conformers of (2*S*,4*R*,10*R*,11*R*,12*R*)-**1**, the *N*-methyl protons and the H-12 were pointing towards opposite directions (distance 4.59 Å) such as the two methylthio groups (Figure 3). Thus pronounced NOE correlation of H-12 and H_3_-21 verified the (2R*,4R*,10R*,11R*,12R*) relative configurations of **1** as shown in Figure 3.

**Figure 4:**
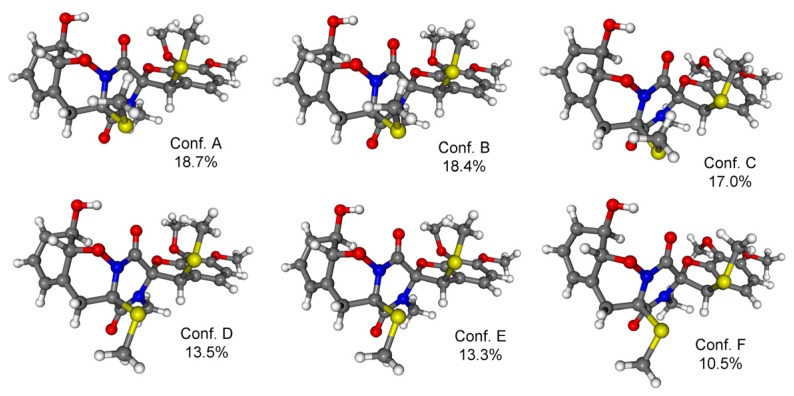
Structures and Boltzmann populations of B97D/TZVP conformers Polarizable Continuum Model (PCM) solvent model for MeCN) of (2*R*,4*R*,10*R*,11*R*,12*R*)-**1**.

**Figure 5:**
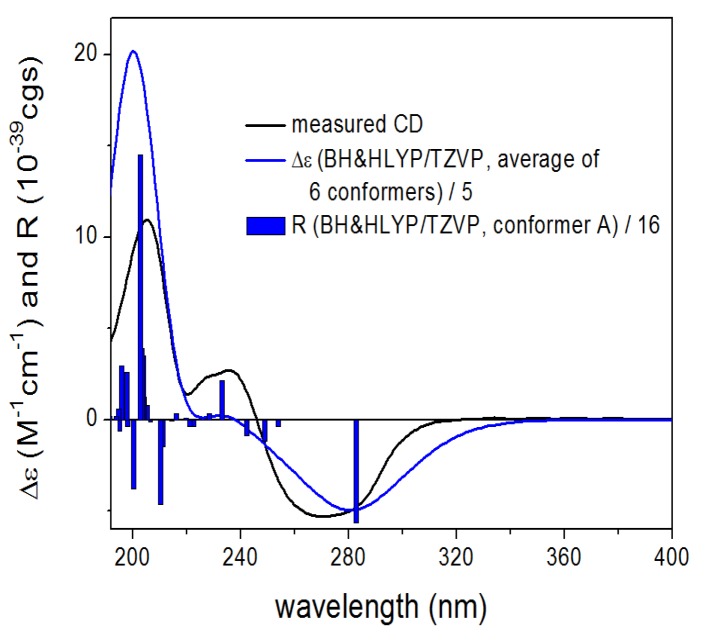
Experimental ECD (**black**) spectrum of **1** compared with BH & HLYP/TZVP ECD spectra (**blue**) of (2*R*,4*R*,10*R*,11*R*,12*R*)-**1** computed for the B97D/TZVP conformers (PCM/MeCN). Bars represent the computed rotational strengths of the lowest-energy conformer.

In order to determine the absolute configuration of **1**, Time-Dependent Density Functional Theory-Electronic Circular Dichroism (TDDFT-ECD) calculations were carried out on the B97D/TZVP conformers of (2*R*,4*R*,10*R*,11*R*,12*R*)**-1** with functionals (B3LYP, BH & HLYP, PBE0) and TZVP basis set (Figure 4). The ECD spectrum of **1** showed a broad negative transition (CE) at 269 nm and positive Cotton effects (CEs) at 237, 226, and 205 nm. The computed TDDFT-ECD spectra of (2*R*,4*R*,10*R*,11*R*,12*R*)-**1** reproduced well the experimental curve with the BH & HLYP/TZVP method giving the best agreement (Figure 5), which allowed determining the absolute configuration as (−)-(2*R*,4*R*,10*R*,11*R*,12*R*) for **1**.

Compound **2** was also obtained as a colorless amorphous powder. It has the molecular formula C_22_H_26_N_2_O_9_S as determined by HR-ESI-MS data. The IR spectrum exhibited broad absorption for OH groups (3275 cm^−1^) as well as for amide chromophores (1678 and 1608 cm^−1^). The ^13^C NMR data (Table 1) exhibited the presence of 22 carbon signals, which were assigned by DEPT and HSQC experiments as four methyls (with two oxygenated, one nitrogenated, and one thiogenated), one methylene, eight methines (with five sp^2^ and three oxygenated sp^3^), and nine quaternary (with two amide carbonyl, five aromatic/olefinic, and two nitrogenated/oxygenated/thiogenated sp^3^) carbons. The ^1^H NMR data (Table 1) along with HSQC experiment displayed resonances characteristic for four methyls (H_3_-19 to H_3_-22), one methylene (H_2_-5), three oxymethines (H-7, H-10, and H-11), and five aromatic/olefinic methines (H-8, H-9, H-12, H-17, and H-18) (Table 1). Comprehensive analysis of the NMR data revealed that the structure of **2** was similar to pretrichodermamide A [1] (Figure 1). The main differences were the presence of signals for additional *N*-methyl (CH_3_-21, δ_C/H_ 35.6/2.91) and *S*-methyl (CH_3_-22, δ_C/H_ 13.9/2.26) in the NMR spectra of **2**, which was proved by the HMBC correlations from H_3_-21 to C-1 and C-2 and from H_3_-22 to C-4 (Figure 2). In addition, two methines (CH-2, δ_C/H_ 59.0/4.41) and (CH-12, δ_C/H_ 45.0/4.49) in the NMR spectra of pretrichodermamide A [1] disappeared in those of **2**, and instead signals (C-2, δ_C_ 129.0) and (CH-12, δ_C/H_ 118.1/7.25) corresponding to a double bond were observed in the NMR spectra of **2**. These changes were supported by the observed HMBC correlations from H-12 to C-3, C-14, and C-18 (Figure 2). Thus the planar structure of **2** could be assigned as shown in Figure 1.

**Figure 6:**
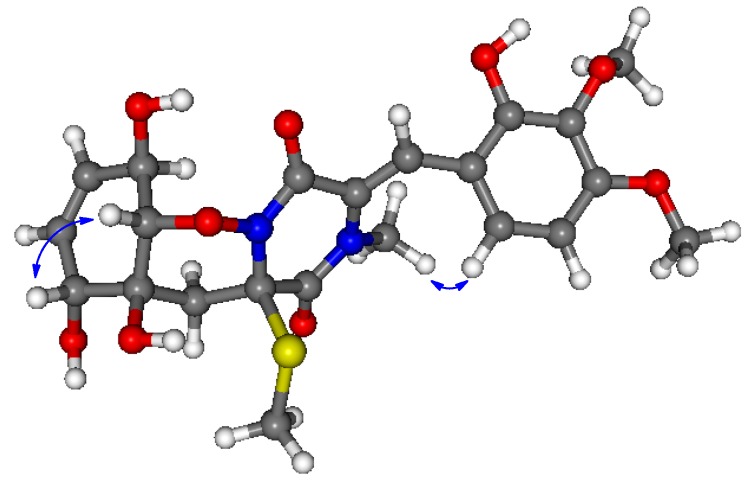
Structure of the lowest-energy B97D/TZVP conformer (PCM solvent model for MeCN) of (4*R*,6*S*,7*R*,10*R*,11*S*)-**2** with the measured NOE correlations.

The (4R*,6S*,7R*,10R*,11S*) relative configuration of **2** was determined by the analysis of vicinal proton-proton coupling constants and NOESY experiments. The coupling constant between H-10 and H-11 (8.2 Hz) suggested that the two protons had *trans*-*diaxial* arrangement, and the NOE correlation between H-7 and H-11 indicated the same orientation of these protons (Figure 6). The chemical shift value of H-12 (δ_H_ 7.25), which is downfield-shifted by the deshielding effect of the C=O of C-3, suggested the (*Z*)-configuration of the C=C double bond at C-2(12), which was further confirmed by the NOE correlation between H_3_-21 and H-18 (Figure 6).

**Figure 7:**
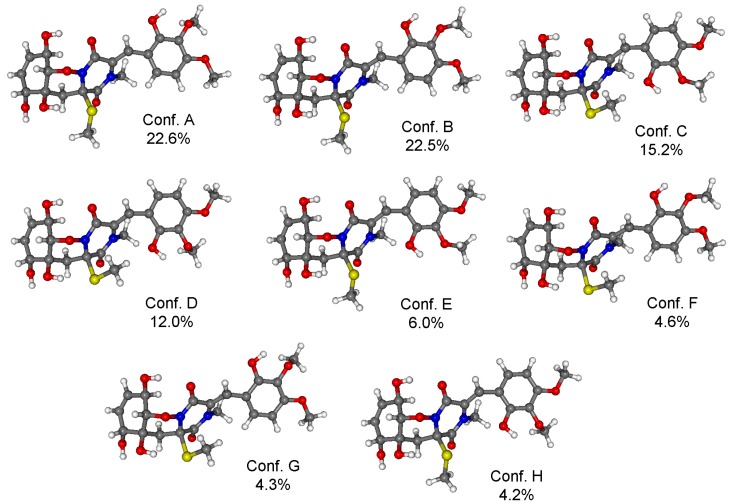
Structures and Boltzmann populations of B97D/TZVP conformers (PCM solvent model for MeCN) of (4*R*, 6*S*, 7*R*, 10*R*,11*S*)-**2**.

**Figure 8:**
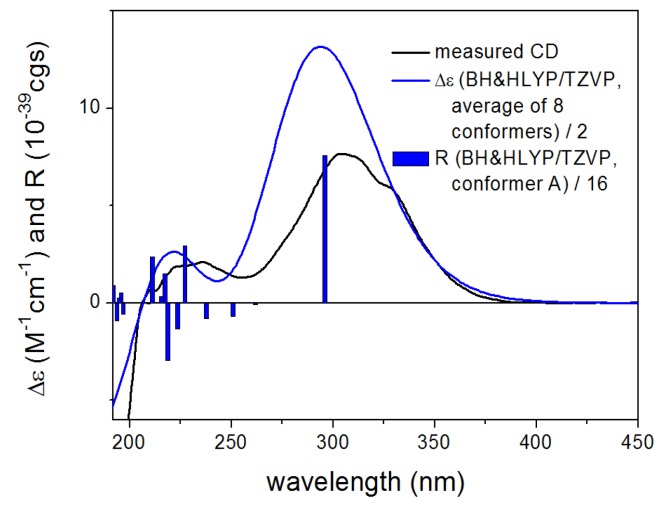
Experimental ECD (**black**) spectrum of **2** compared with BH & HLYP/TZVP ECD spectra (**blue**) of (4*R*,6*S*,7*R*,10*R*,11*S*)-**2** computed for the B97D/TZVP conformers (PCM/MeCN). Bars represent the computed rotational strengths of the lowest-energy conformer.

For the configurational assignment of **2**, conformational analysis and TDDFT-ECD calculations of the solution conformers were carried out. The B97D/TZVP reoptimization of the initial Merck Molecular Force Field (MMFF) conformers of (4*R*,6*S*,7*R*,10*R*,11*S*)-**2** afforded eight slightly different conformers, in which the equatorial 10-OH was hydrogen-bonded to the C-3 carbonyl oxygen, while 6-OH to the 4-SMe group fixing the chair conformation of the 1,2-oxazine ring (Figure 7). The ECD spectrum of **2** was dominated by the intense positive CE at 304 nm accompanied with shoulders and weaker positive CEs at 236, 220, and 207 nm. The TDDFT-ECD spectra of (4*R*,6*S*,7*R*,10*R*,11*S*)-**2** reproduced well the experimental ECD transitions (Figure 8) and thus the absolute configuration was determined as (+)-(4*R*,6*S*,7*R*,10*R*,11*S*) and the trivial name 6,7-dihydroxy-*seco*-peniciadametizine A was assigned to **2**.

In addition to the new compounds **1** and **2**, two known secondary metabolites, brasiliamide A (**3**) [10] and viridicatumtoxin (**4**) [11] (Figure 1), were also isolated, and their structures were elucidated by comparing the NMR data with those of literature reports.

**Scheme 1:**
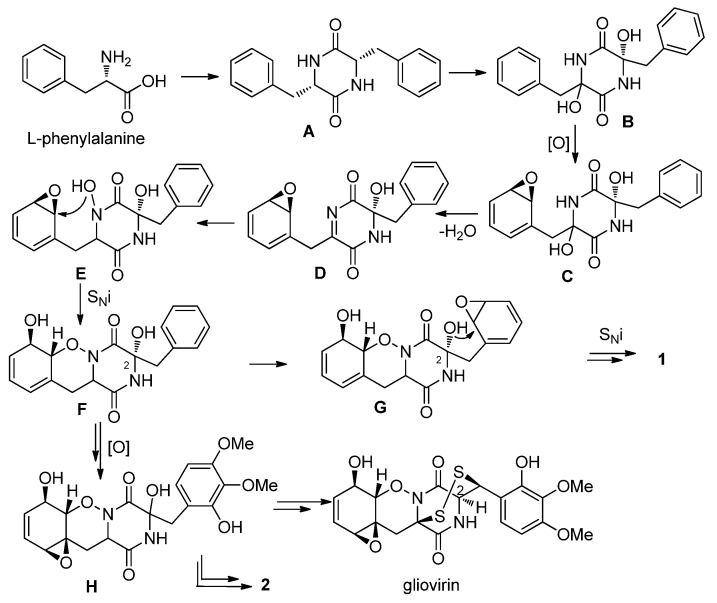
Proposed biosynthetic pathway for **1** and **2**.

Peniciadametizine A (**1**) represents a new member of the gliovirin family whose sulfide functionalities are attached to the α- and β-positions of the degraded amino acid residues. Particularly, the spiro[furan-2,7*'*-pyrazino[1,2-*b*][1,2]oxazine] skeleton of **1** has not been reported yet among natural products. The isolation of compounds **1** and **2** provided further insight into the biosynthesis of thiodiketopiperazine derivatives, which has been recently studied by several research groups [12]. The biosynthesis of these derivatives starts with the dimerization of L-phenylalanine followed by bishydroxylation to afford intermediate B, the hydroxyl groups of which are usually replaced in the following step(s) by thiol groups (Scheme 1). However, the structures of **1** and **2** suggest a different pathway, in which the two halves of the diketopiperazine moiety follow different sequence of reactions. Upon epoxidation of the phenyl ring one of the hydroxyl groups is lost by dehydration to produce an imine group, the nitrogen of which is then hydroxylated (Scheme 1). The isolation of a related *N*-hydroxyl intermediate was reported earlier in targeted gene-deletion study [13]. The *N*-hydroxyl group opens the epoxide ring forming a 1,2-oxazine ring (**E**→**F**), while the epoxidation of the Δ^6^ double bond provides intermediate **H**, the precursor of both gliovirin and compound **2**. If the hydroxyl group at C-2 of intermediate F is involved in an epoxide ring-opening reaction, a dihydrobenzo[*b*]furan moiety is produced (**F**→**G**→**1**), the further transformation of which affords compound **1**. It is noteworthy that gliovirin has (2*S*) absolute configuration as confirmed by X-ray analysis [14], which means that the original (*S*) configuration of intermediate **A** was inverted during the biosynthetic scheme.

Compounds **1** and **2** were tested for cytotoxic activities against nine human tumor cell lines (DU145, HeLa, HepG2, MA, MCF-7, SGC-7901, SW1990, SW480, and U251). None of them showed potent activity (IC_50_ > 10 μM). However, they exhibited weak brine shrimp lethality against *Artemia salina*, with lethal ratio of 45.5 and 62.4% at the concentration of 100 μg/mL (Table S1 in the Supplementary file). Compounds **1** and **2** were also assayed for antimicrobial activities against human- and aqua-pathogenic bacteria (*Aeromonas hydrophilia*, *Edwardsiella tarda*, *Escherichia coli*, *Staphyloccocus aureus*, *Vibrio alginolyticus*, *V. anguillarum*, *V. parahaemolyticus*, and *V. harveyi*), and plant pathogenic fungi (Alternaria brassicae, Colletotrichum gloeosporioides, Fusarium graminearum, and Gaeumannomyces graminis), and they exhibited selective activities against *A. brassicae* with MIC values of 4.0 and 32.0 μg/mL, respectively, compared to amphotericin B with an MIC value of 1.0 μg/mL.

## 3. Experimental Section

### 3.1. General

Optical rotations were measured on an Optical Activity AA-55 polarimeter (Optical Activity Ltd., Cambridgeshire, UK). UV spectra were measured on a PuXi TU-1810 UV-visible spectrophotometer (PuXi Analytical Instrument Co. Ltd, Beijing, China). CD spectra were acquired on a J-810 spectropolarimeter (JASCO International Co. Ltd, Tokyo, Japan). IR spectra were obtained on a Thermo Scientific Nicolet iS10 spectrophotometer (Thermo Fisher, Waltham, MA, USA). The ^1^H, ^13^C, and 2D NMR spectra were acquired using a Bruker Avance 500 spectrometers (Bruker Biospin Group, Karlsruhe, Germany). Low- and high-resolution MS data were obtained on a Q-TOF Ultima Global GAA076 LC mass spectrometer (Waters Asia, Ltd., Singapore). Semi-preparative HPLC was performed using a Dionex UltiMate U3000 system (Dionex Corporation, Sunnyvale, CA, USA) with an Agilent Prep RP-18 column (21.2 × 250 mm, 10 μm) under UV detection. Column chromatography (CC) was performed with silica gel (200–300 mesh, Qingdao Haiyang Chemical Factory, Qingdao, China), Lobar LiChroprep RP-18 (40–60 μm, Merck, Darmstadt, Germany), and Sephadex LH-20 (18–110 μm, Merck, Darmstadt, Germany). Solvents for extraction and purification were distilled prior to use.

### 3.2. Fungal Material

The fungus *Penicillium adametzioides* AS-53 was isolated from the fresh tissue of an unidentified marine sponge that was collected from Wenchang, Hainan, China, in September 2011. The fungus was identified by analysis of its Internal Transcribed Spacer (ITS) region of the rDNA, as described in our previous report [15]. The sequence data of 5.8S rDNA and ITS regions derived from the fungal strain have been submitted to and deposited at GenBank with accession no. KJ906543. The nucleotide BLAST search result showed that the sequence was the most similar (99%) to the sequence of *P. adametzioides* strain DTO115I8 (compared to accession no. KC773824.1). The strain is preserved at the Key Laboratory of Experimental Marine Biology, Institute of Oceanology, Chinese Academy of Sciences.

### 3.3. Fermentation

For chemical investigation, the fresh mycelia of *P. adametzioides* AS-53 were grown on potato dextrose agar (PDA) medium at 28 °C for 5 days and were then inoculated into 1 L conical flask containing 300 mL of potato-dextrose broth (PDB) medium (1000 mL sea water, 20 g glucose, 5 g peptone, and 3 g yeast extract, pH (6.5–7.0)) for 30 days at room temperature.

### 3.4. Extraction and Isolation

All of the fermented cultures (60 flasks, 18 L) were filtered to separate the broth from the mycelia. The former was extracted four times with ethyl acetate (EtOAc), while the latter was extracted four times with mixture of 80% acetone and 20% H_2_O. The acetone solution was evaporated under reduced pressure to afford an aqueous solution, which was then extracted four times with EtOAc. Because the TLC and HPLC profiles of the two EtOAc solutions were almost identical, they were combined and concentrated under reduced pressure to give an extract (37 g), which was fractionated by silica gel vacuum liquid chromatography (VLC) using different solvents of increasing polarity from petroleum ether (PE) to MeOH to yield 8 fractions (Frs. 1–8) based on TLC analysis. Fr. 4 (3.6 g) was further purified by reversed-phase column chromatography (CC) over Lobar LiChroprep RP-18 with a MeOH–H_2_O gradient (from 10:90 to 100:0) to yield 10 subfractions. Fr. 4-5 (MeOH–H_2_O 1:1, 480 mg) was further fractionated by CC on silica gel eluting with CHCl_3_–MeOH (from 100:1 to 10:1) to afford five subfractions (from Fr. 4-5-1 to Fr. 4-5-5). Fr. 4-5-1 (45 mg) was then purified by preparative TLC (CHCl_3_–acetone, 2:1) to give compound **1** (4.5 mg, R_f_ = 0.5). Fr. 4-8 (MeOH–H_2_O 4:1, 180 mg) was further fractionated by CC on silica gel eluting with CHCl_3_–MeOH (from 150:1 to 20:1) to afford compound **4** (8.3 mg). Fr. 5 (4.4 g) was further purified by reversed-phase CC over Lobar LiChroprep RP-18 (Merck, Darmstadt, Germany) with a MeOH–H_2_O gradient (from 10:90 to 100:0) to yield 10 subfractions. Fr. 5**-**6 (MeOH–H_2_O 3:2, 350 mg) was further fractionated by CC on silica gel eluting with CHCl_3_–MeOH (from 100:1 to 10:1) to afford five subfrations, one of which (Fr. 5**-**6-4, 45 mg) was purified by CC on Sephadex LH-20 (MeOH), to yield compound **3** (8.5 mg). Fr. 6 (4.7 g) was further purified by reversed-phase CC over Lobar LiChroprep RP-18 with a MeOH–H_2_O gradient (from 10:90 to 100:0) to yield 10 subfractions. Fr. 6**-**4 (MeOH–H_2_O 2:3, 850 mg) was further fractionated by CC on silica gel eluting with CHCl_3_–MeOH (from 100:1 to 10:1) to afford four subfractions (from Fr. 6-4-1 to Fr. 6-4-4). Fr. 6-4-3 (140 mg) was purified by semi-preparative HPLC eluting with 45% aqueous MeOH to afford **2** (6.2 mg, *t_R_* = 25.155 min).

Peniciadametizine A (**1**): colorless amorphous powder; ${\left[\alpha \right]}_{D}^{25}$ −25.0 (*c* 0.16, MeOH); UV (MeOH) λ_max_ (log ε) 206 (4.14), 276 (3.14) nm; ECD (MeCN) λ_max_ (Äε) 205 (11.42), 226sh (2.37), 237 (2.86), 269 (−5.31) nm; IR *v*_max_ 3334, 2918, 2849, 1679, 1602, 1502, 1462, 1365, 1286, 1091, 1053 cm^−1^; ^1^H and ^13^C NMR data, see Table 1; TOF-ESI-MS *m*/*z* 545 [M + K]^+^, 529 [M + Na]^+^; HR-ESI-MS *m*/*z* 529.1060 [M + Na]^+^ (calcd for C_23_H_2__6_N_2_O_7_S_2_Na, 529.1074).

Peniciadametizine B (**2**): colorless solid; ${\left[\alpha \right]}_{D}^{25}$ +150.0 (*c* 0.06, MeOH); UV (MeOH) λ_max_ (log ε) 202 (4.17), 256 (3.36), 322 (3.63) nm; ECD (MeCN) λ_max_ (Äε) negative below 206 nm, 207 (1.25), 220 (1.98), 236 (2.23), 304 (7.74), 312 sh (7.51), 328sh (6.04) nm; IR *v*_max_ 3275, 2919, 2850, 1678, 1608, 1504, 1463, 1361, 1288, 1098, 1042 cm^−1^; ^1^H and ^13^C NMR data, see Table 1; TOF-ESI-MS *m*/*z* 989 [2M + H]^+^, 533 [M + K]^+^, 517 [M + Na]^+^, 495 [M + H]^+^; HR-ESI-MS *m*/*z* 495.1428 [M + H]^+^ (calcd for C_22_H_27_O_9_N_2_S^+^, 495.1432).

### 3.5. Computational Section

Mixed torsional/low mode conformational searches were carried out by means of the Macromodel 9.9.223 [16] software using Merck Molecular Force Field (MMFF) (Schrödinger, LLC, Portland, OR, USA) with implicit solvent model for chloroform applying a 21 kJ/mol energy window. Geometry reoptimizations of the resultant conformers (B3LYP/6-31G(d) level in vacuo and B97D/TZVP [17,18] level with PCM solvent model for MeCN) and TDDFT calculations were performed with Gaussian 09 [19] using various functionals (B3LYP, BH & HLYP, PBE0) and TZVP basis set. ECD spectra were generated as the sum of Gaussians [20] with 3600 and 4200 cm^−1^ half-height width (corresponding to *ca.* 28 and 33 nm at 280 nm), using dipole-velocity computed rotational strengths. Boltzmann distributions were estimated from the Zero Point Vibrational Energy ZPVE corrected B3LYP/6-31G(d) energies in the gas-phase calculations and B97D/TZVP energies in the solvent model calculations. The MOLEKEL [21] software package was used for visualization of the results.

### 3.6. Brine Shrimp Assay

Evaluations of the isolated compounds for brine shrimp (*A. salina*) lethality were determined as described previously [22]. Colchicine was used as a positive control.

### 3.7. Antimicrobial Assays

The antimicrobial activities against human- and aqua-pathogenic bacteria (*A. hydrophilia*, *E. coli*, *E. tarda*, *S. aureus*, *V. alginolyticus*, *V. anguillarum*, *V. harveyi*, and *V. parahaemolyticus*) as well as four plant pathogenic fungi (*A. brassicae*, *C. gloeosprioides*, *F. graminearum*, and *G. graminis*) of the new compounds **1** and **2** were evaluated using the methods described previously [22]. Chloramphenicol and amphotericin B were used as positive controls against bacteria and fungi, respectively.

### 3.8. Cytotoxicity Assays

The cytotoxic activities of the new compounds **1** and **2** against 9 tumor cell lines including human prostate carcinoma cell line (Du145), human cervixl carcinoma cell line (HeLa), human liver hepatocellular cells (HepG2), mouse Leydig tumor cell line (MA), human breast carcinoma cell line (MCF-7), human gastric carcinoma cell line (SGC-7901), human pancreatic cancer cell line (SW1990), human colon carcinoma cancer (SW480), and human glioma cells (U251) were determined according to previously reported methods [23,24].

## 4. Conclusions

In conclusion, two new diketopiperazines, peniciadametizines A and B (**1** and **2**), along with two known secondary metabolites, brasiliamide A (**3**) and viridicatumtoxin (**4**) were isolated and characterized from *P. adametzioides* AS-53, a fungal strain obtained from an unidentified marine sponge. To our knowledge, compound **1** possesses a spiro[furan-2,7*'*-pyrazino[1,2-*b*][1,2]oxazine] skeleton, which has not been described yet among natural products. Both compounds exhibited selective activity against the pathogenic fungus *A. brassicae*.

## Supplementary Files

Supplementary File 1
