# Supplementary material for: Peniciadametizine A, a Dithiodiketopiperazine with a Unique Spiro[furan-2,7'-pyrazino[1,2-b][1,2]oxazine] Skeleton, and a Related Analogue, Peniciadametizine B, from the Marine Sponge-Derived Fungus Penicillium adametzioides
Source: Mar Drugs. 2015 Jun 5;13(6):3640–52. doi: 10.3390/md13063640 (PMC4483649; doi:10.3390/md13063640)
Supplement: Supplementary File 1 [file marinedrugs-13-03640-s001.pdf]

Supplementary Information

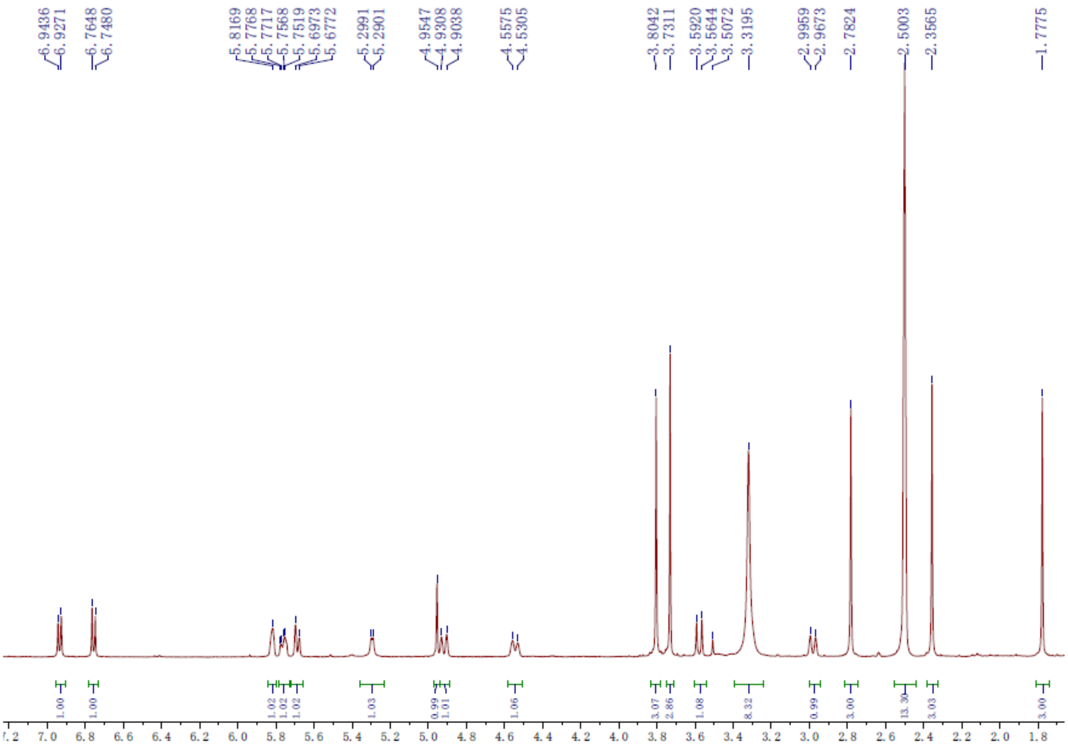

Figure S1. ^{1}H NMR (500 MHz, DMSO-d_6) spectrum of compound **1**.

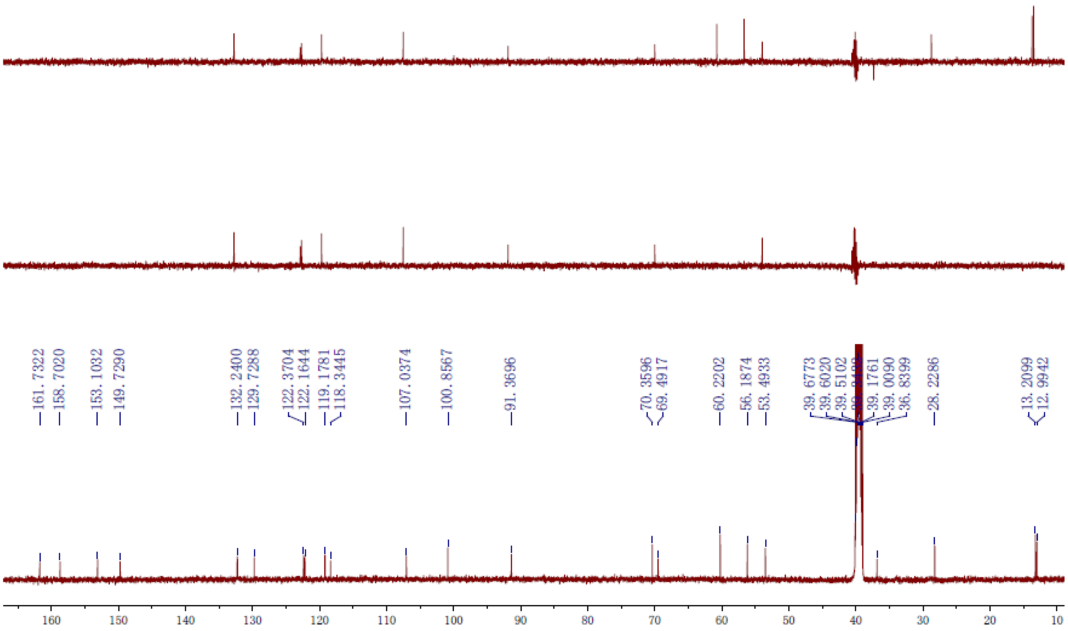

Figure S2. ^{13}C NMR (125 MHz, DMSO-d_6) and DEPT spectra of compound **1**.

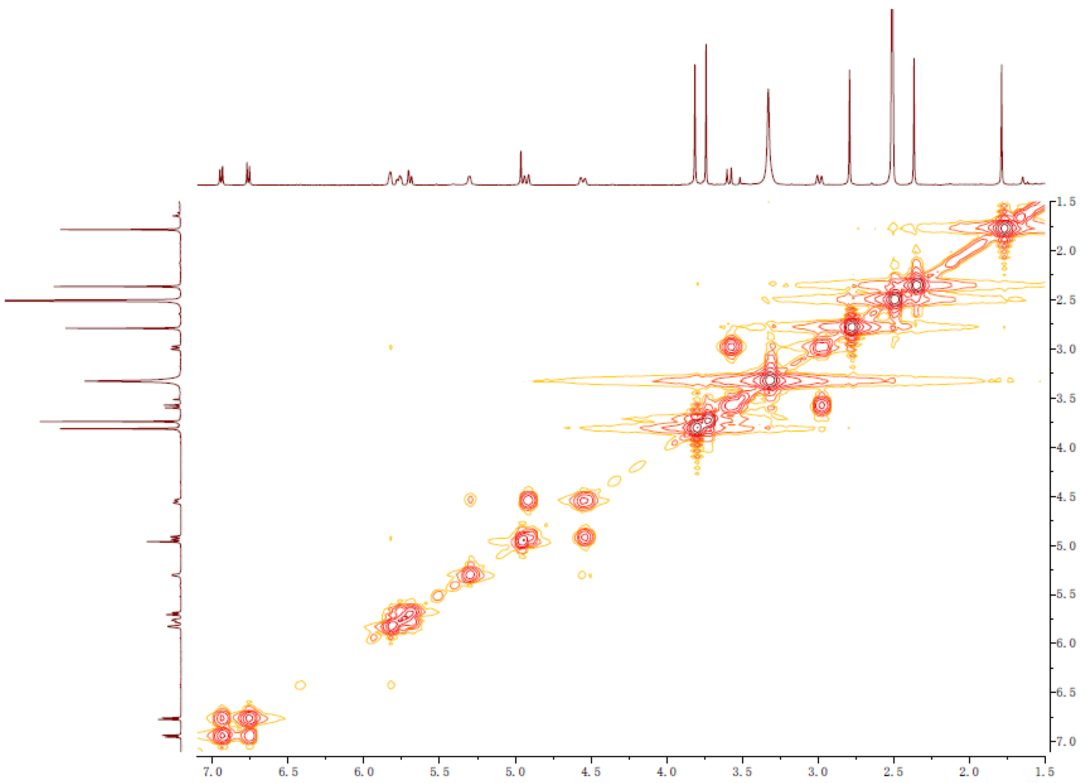

Figure S3. COSY spectrum of compound **1**.

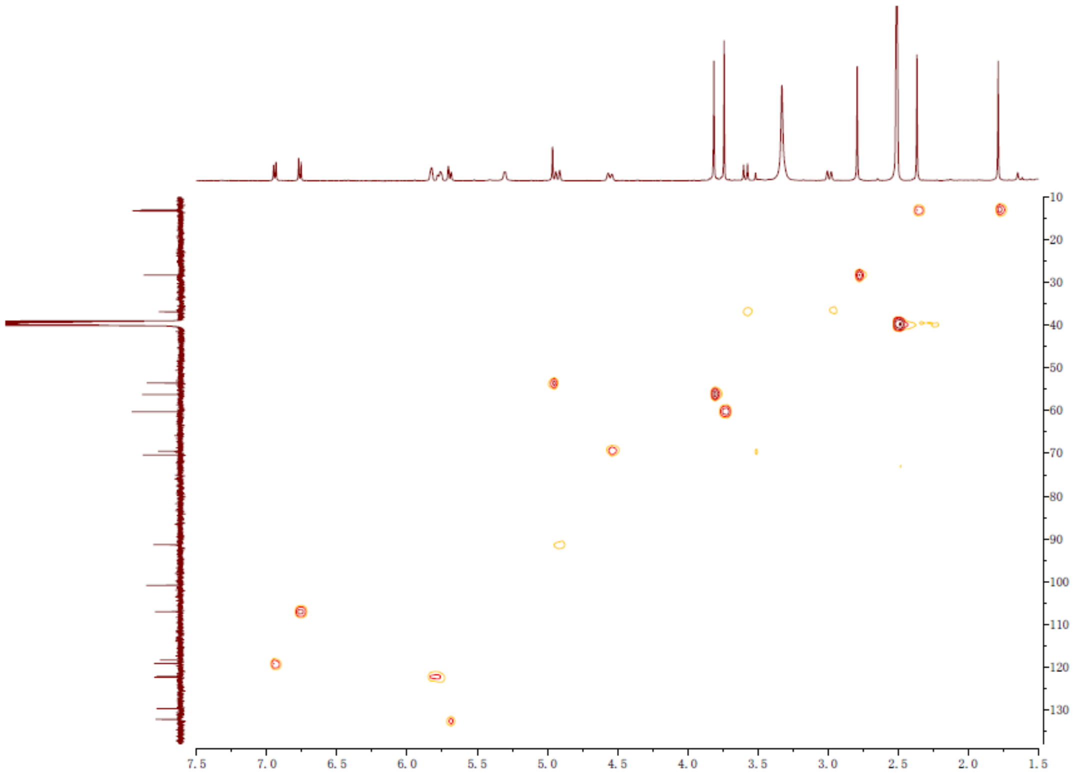

Figure S4. HSQC spectrum of compound **1**.

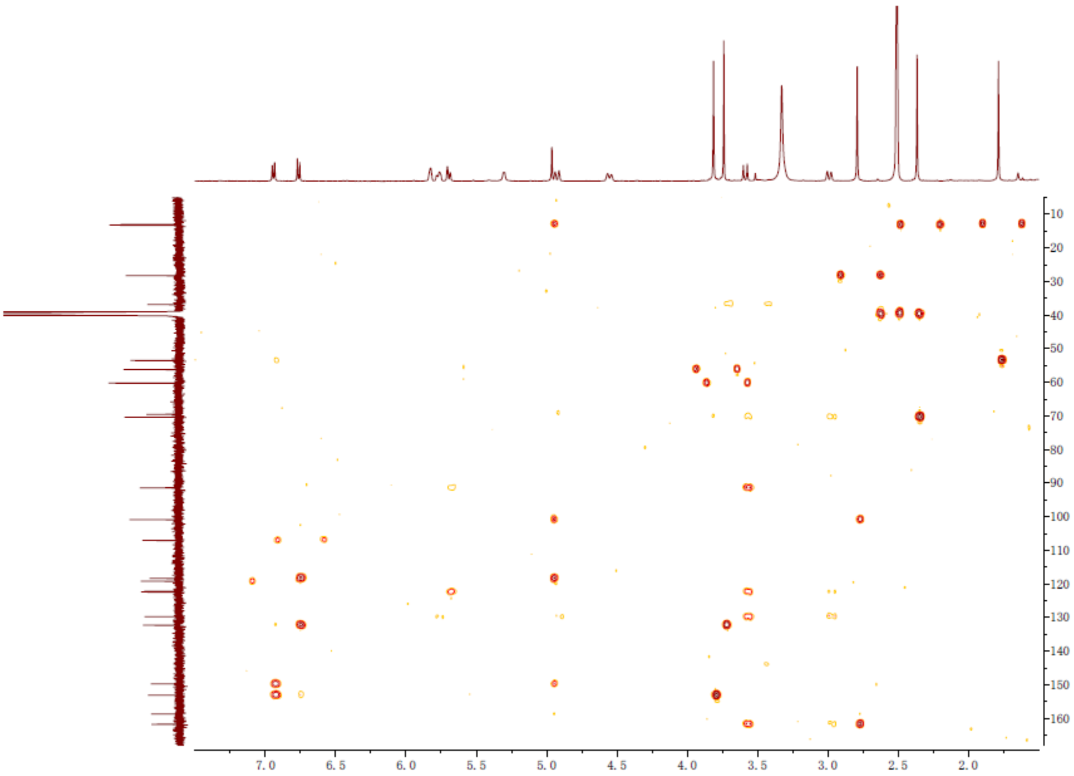

Figure S5. HMBC spectrum of compound **1**.

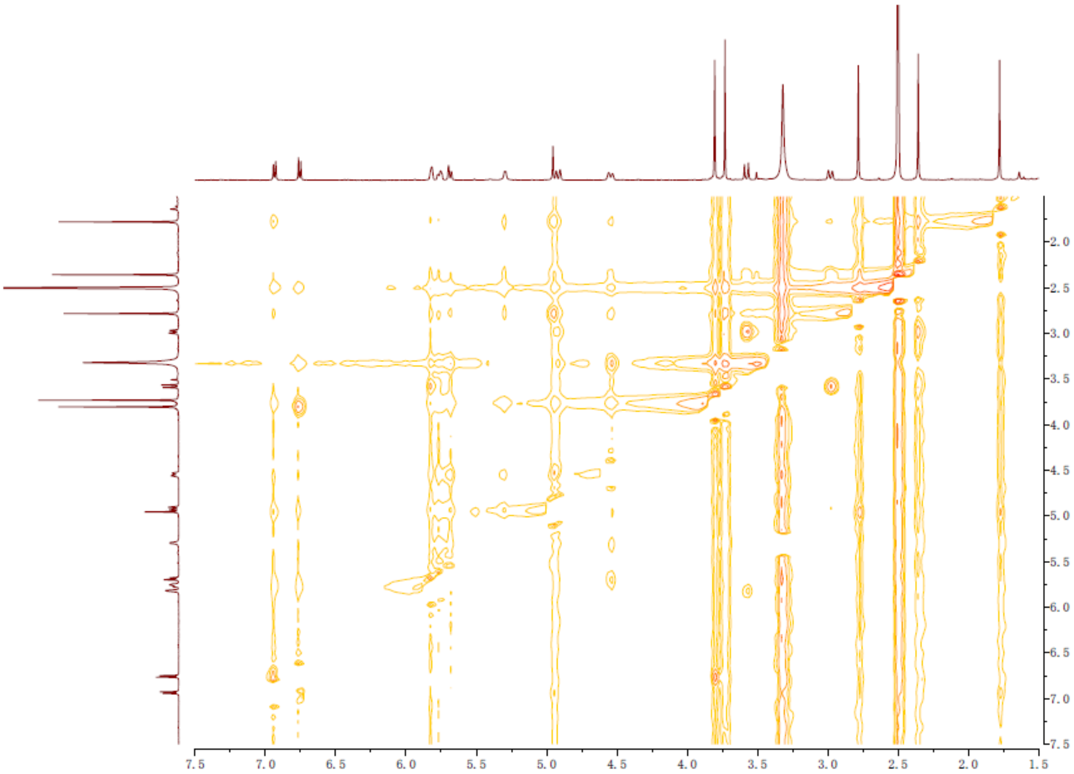

Figure S6. TOF-ESI-MS spectrum of compound **1**.

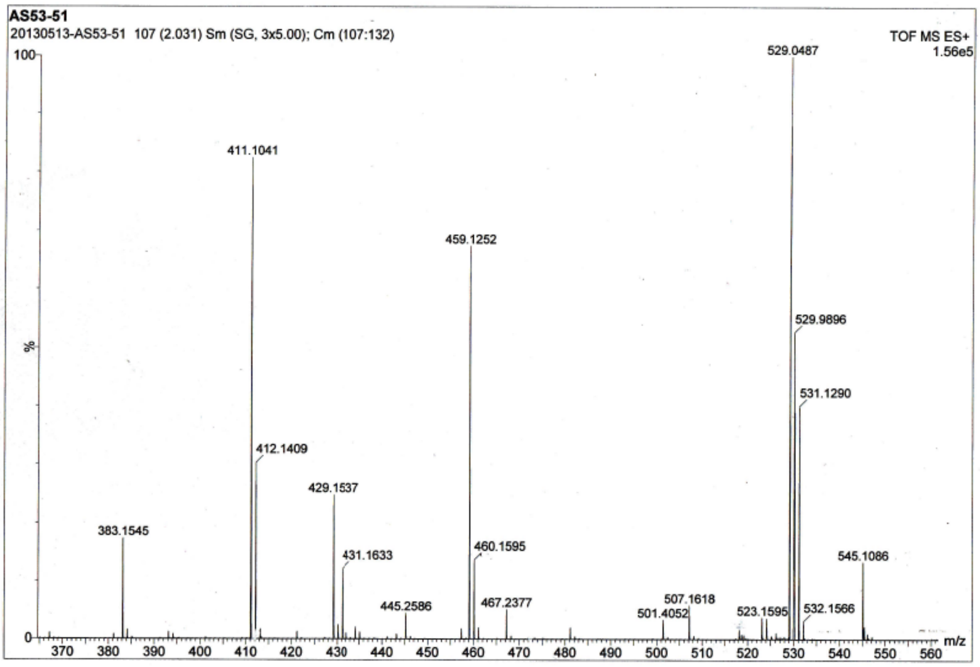

Figure S7. NOESY spectrum of compound **1**.

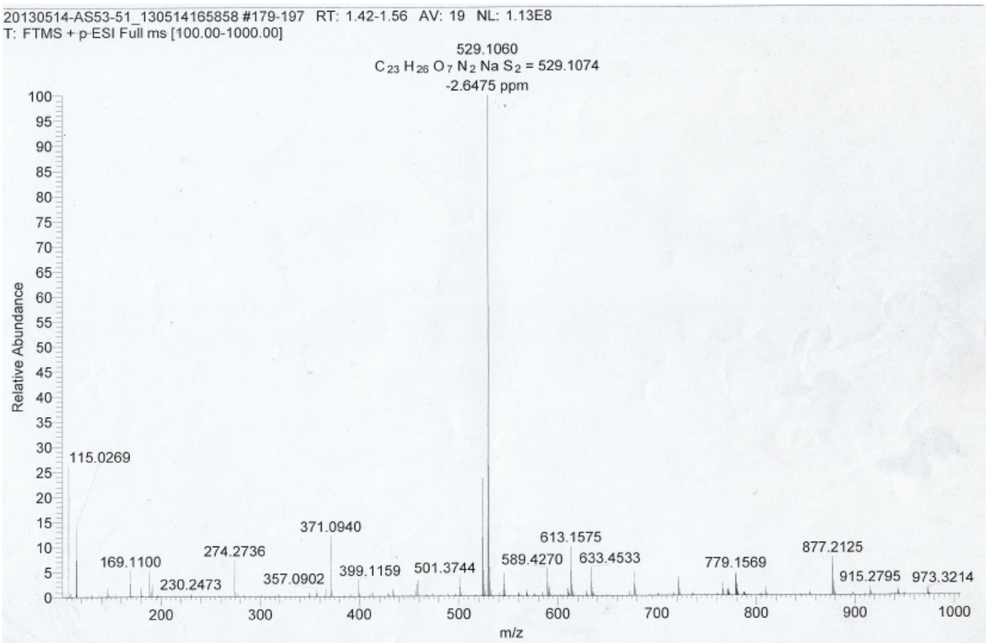

Figure S8. HR-ESI-MS spectrum of compound **1**.

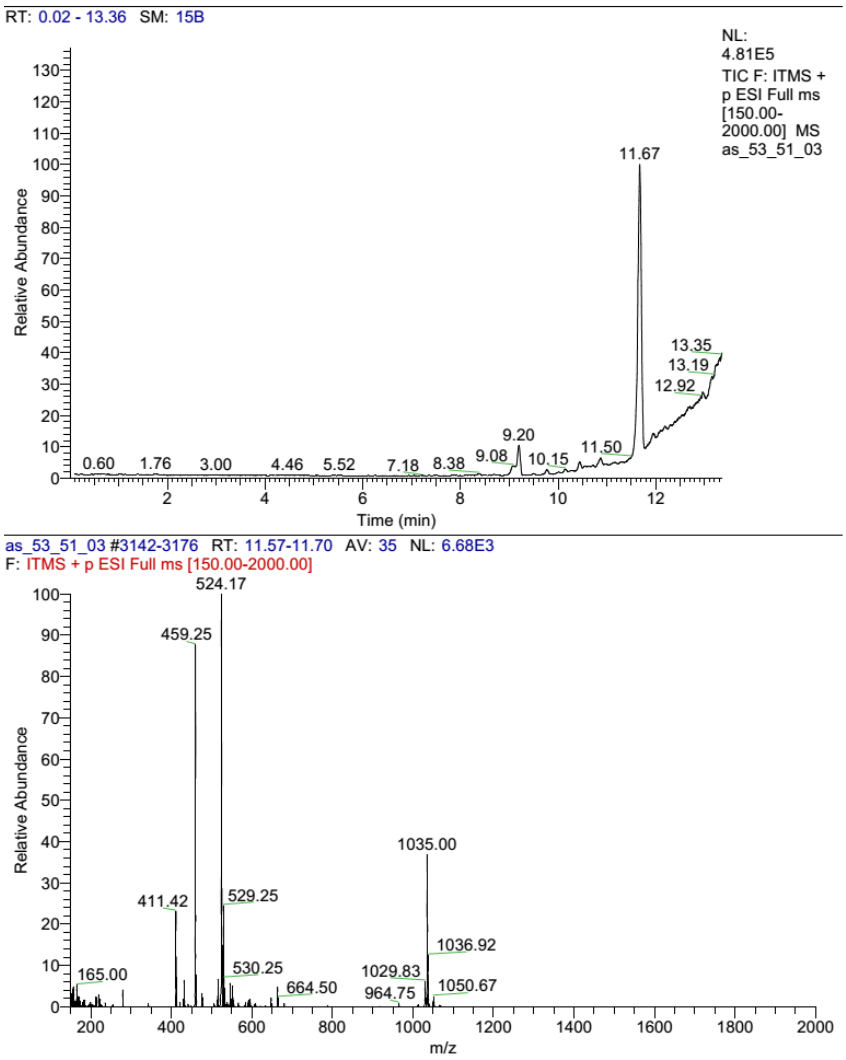

Figure S9. HPLC-UV chromatogram and HPLC-MS trace of **1**. Base peak (524.17): $[M + H_2O]^+$, 529: $[M + Na]^+$, 1035: $[2M + Na]^+$.

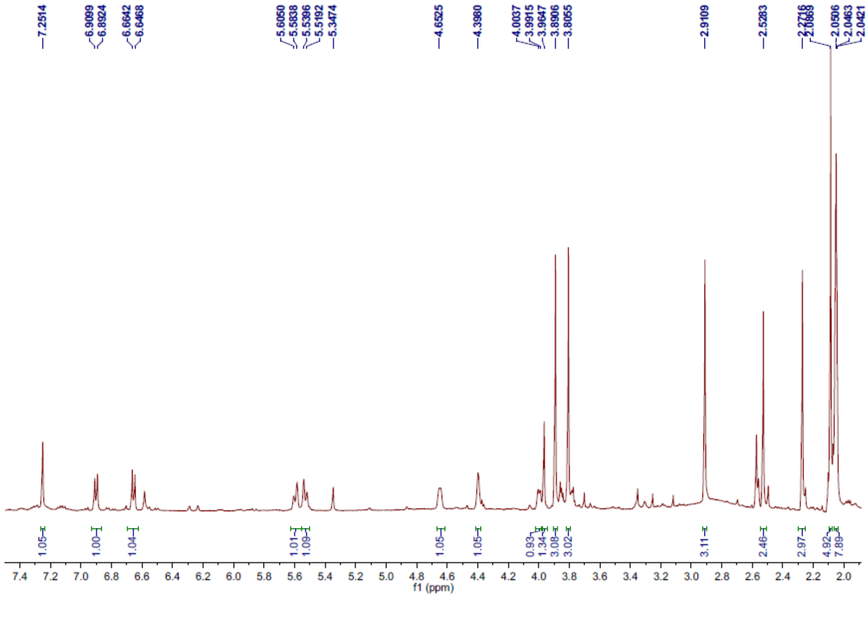

Figure S10. ^{1}H NMR (500 MHz, acetone-d_6) spectrum of compound **2**.

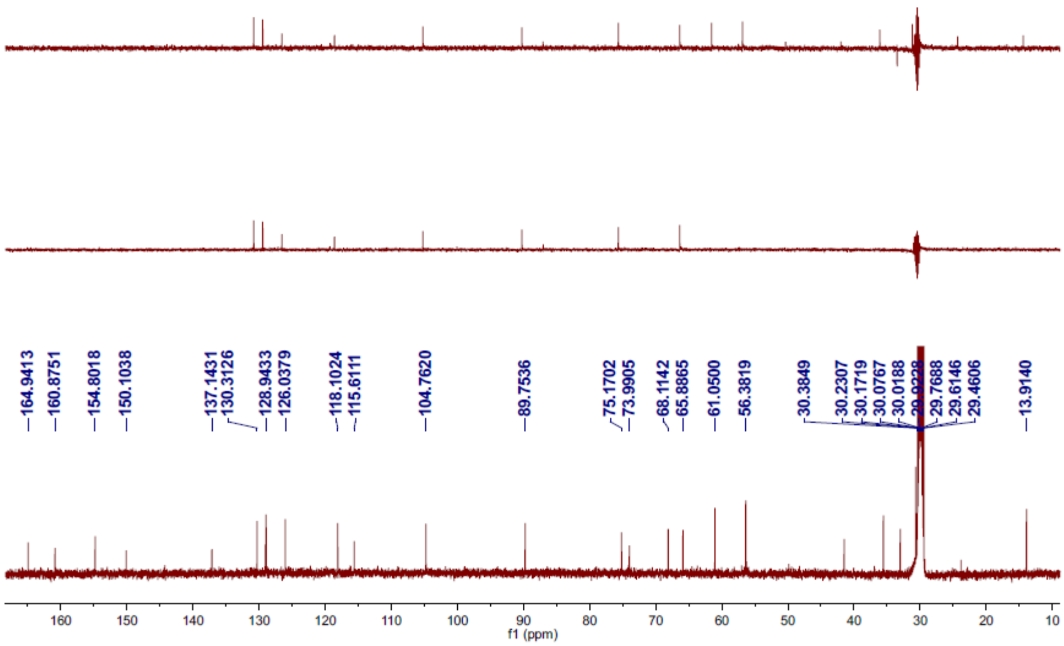

Figure S11. ^{13}C NMR (125 MHz, acetone-d_6) and DEPT spectra of compound **2**.

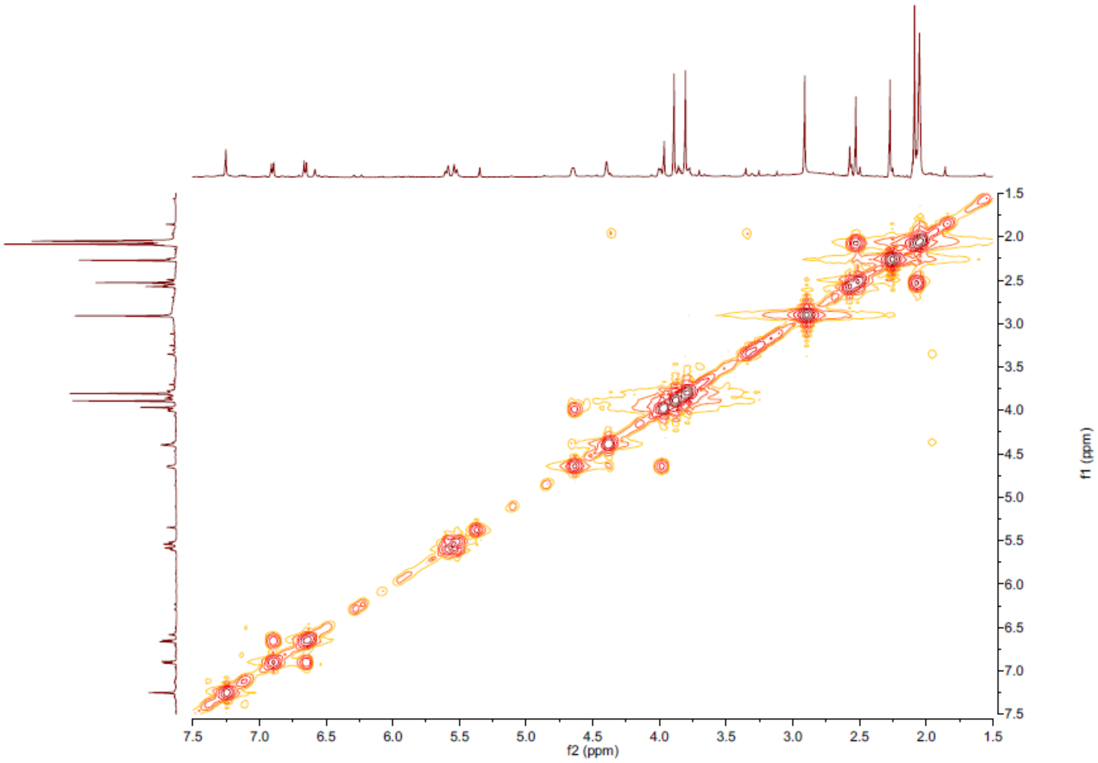

Figure S12. COSY spectrum of compound **2**.

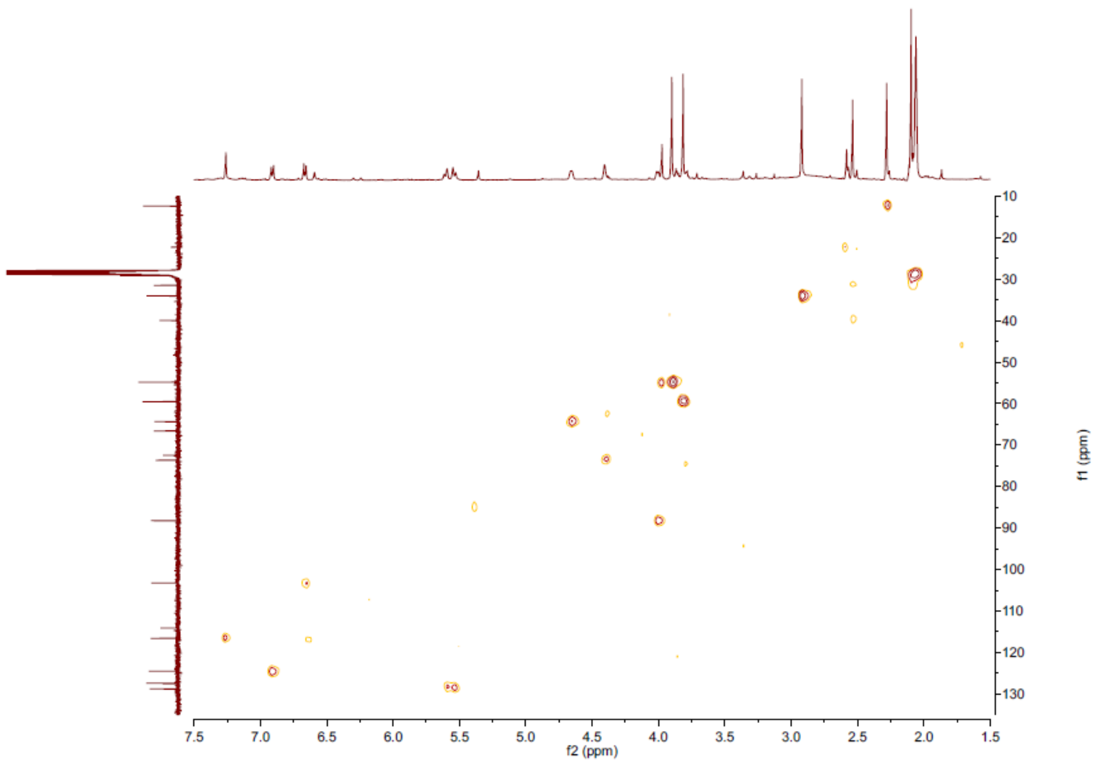

Figure S13. HSQC spectrum of compound **2**.

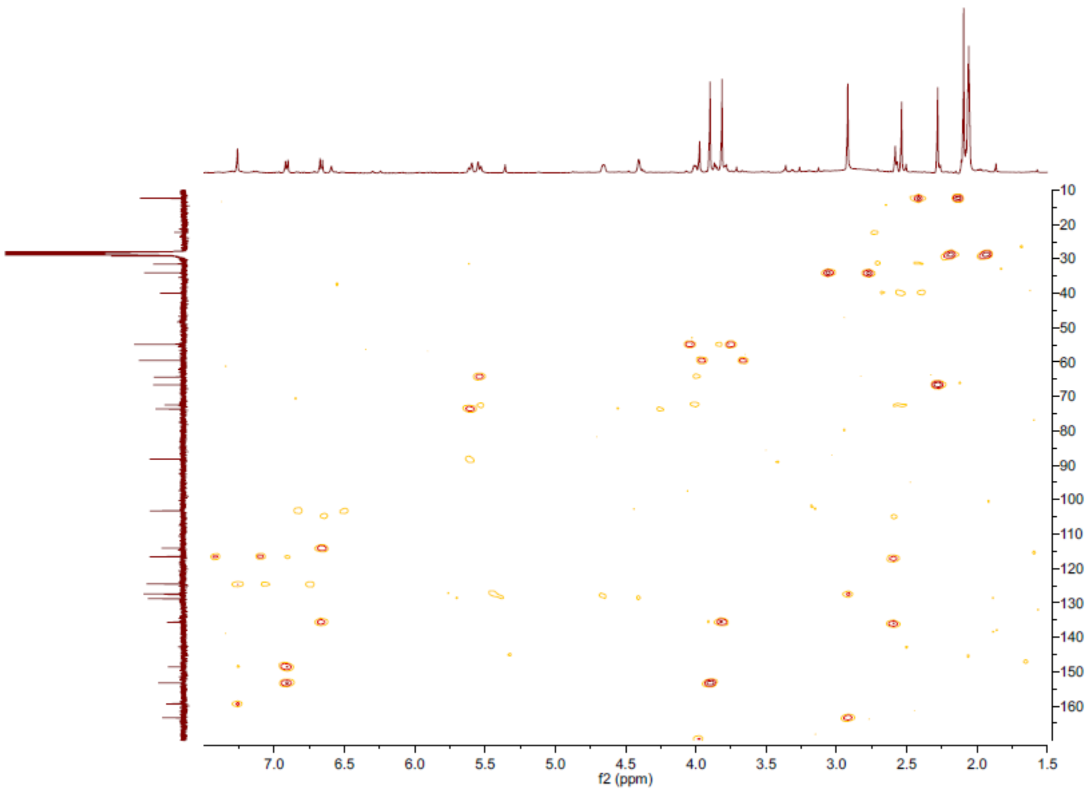

Figure S14. HMBC spectrum of compound **2**.

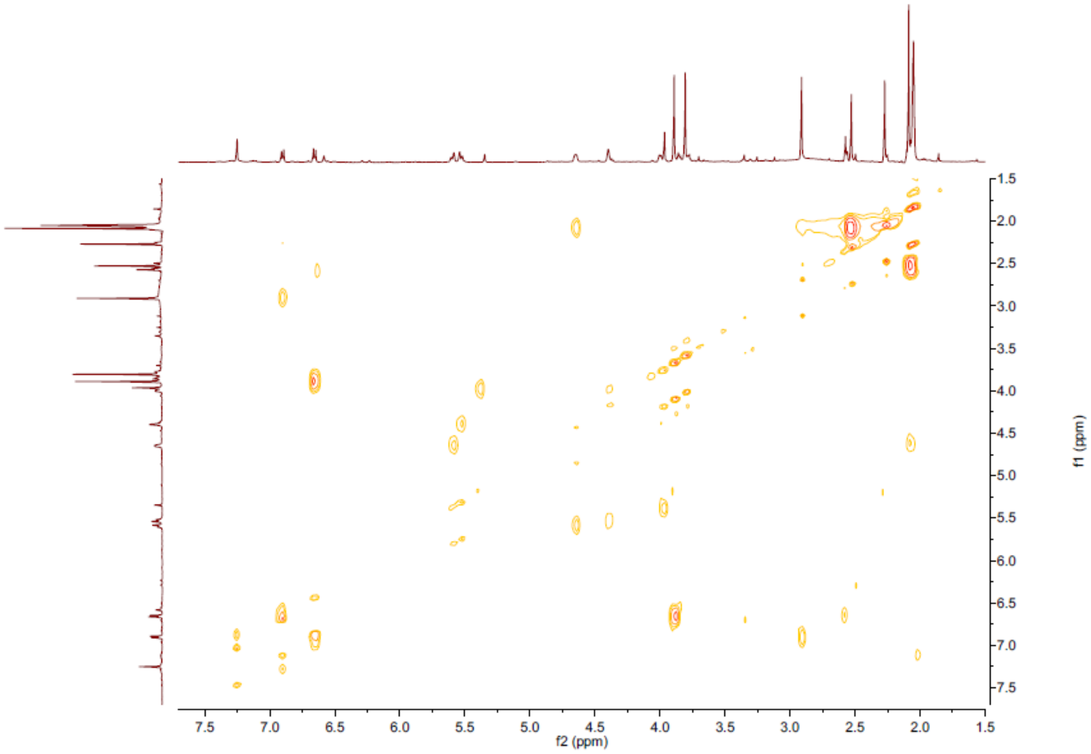

Figure S15. NOESY spectrum of compound **2**.

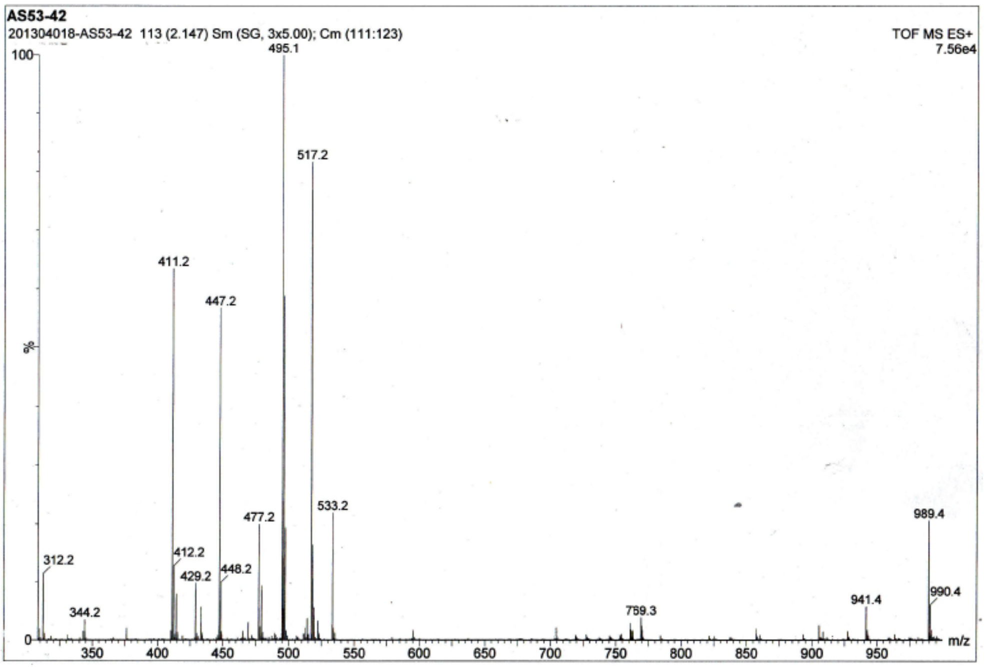

Figure S16. TOF-ESI-MS spectrum of compound **2**.

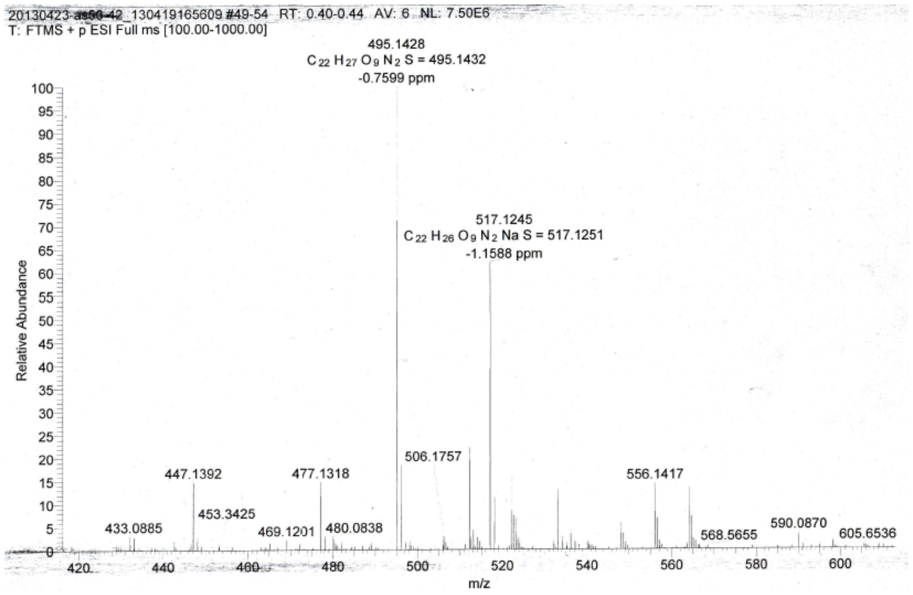

Figure S17. HR-ESI-MS spectrum of compound **2**.

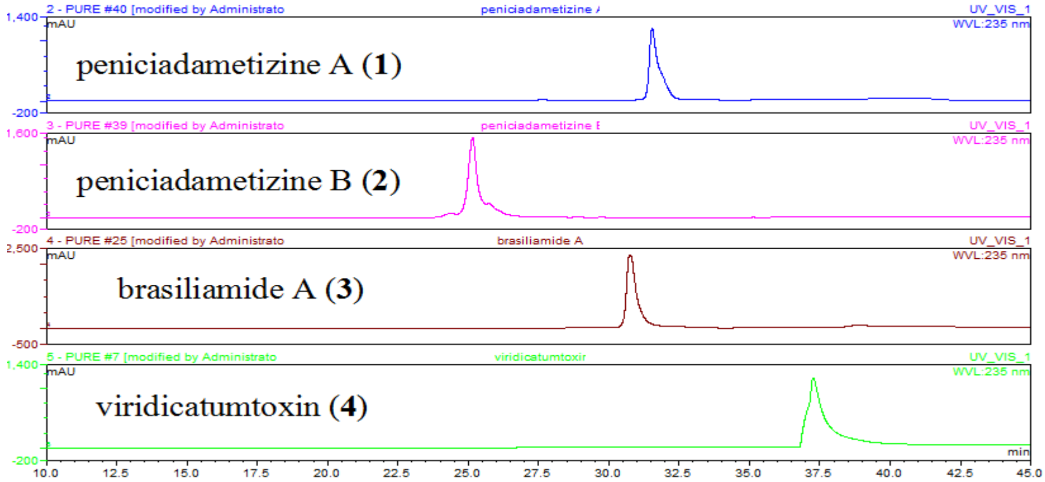

Figure S18. HPLC profiles of compounds **1**–**4** (Dionex Acclaim ODS column, 4.6 × 250 mm, 5 µm; gradient (MeOH and H_2O, 1 mL/min): 0~5 min, 10% MeOH, 5~35 min, 10% to 100% MeOH, 35~45 min, 100% MeOH, 45~50 min, 100% to 10% MeOH, 50~60 min, 10% MeOH).

Table S1. Brine shrimp lethality against *Artemia salina* of compounds **1** and **2**.

Concentration (µg·mL^{-1})	100	50	25	12.5	6.25
**1**	45.5	29.6	28.7	24.9	21.1
**2**	62.4	36.5	32.8	30.4	22.9
colchicine	91.0	85.5	78.0	72.3	72.3
GITC	100	100	96.3	93.3	69.0

Positive controls: colchicine and 2,3,4,6-tetra-*O*-acetyl-β-D-glucopyranosyl isothiocyanate (GITC).

© 2015 by the authors; licensee MDPI, Basel, Switzerland. This article is an open access article distributed under the terms and conditions of the Creative Commons Attribution license (http://creativecommons.org/licenses/by/4.0/).
